# Prevention of non-suicidal self-injury: construction and validation of educational material*

**DOI:** 10.1590/1518-8345.6265.3735

**Published:** 2022-11-07

**Authors:** Aline Conceição Silva, Adriana Inocenti Miasso, Alisson Araújo, Teresa Maria Mendes Dinis de Andrade Barroso, José Carlos Pereira dos Santos, Kelly Graziani Giacchero Vedana

**Affiliations:** 1 Universidade de São Paulo, Escola de Enfermagem de Ribeirão Preto, PAHO/WHO Collaborating Centre for Nursing Research Development, Ribeirão Preto, SP, Brazil.; 3 Universidade Federal de São João del-Rei, Campus Centro Oeste Dona Lindu, Divinópolis, MG, Brazil.; 4 Escola Superior de Enfermagem de Coimbra, Unidade de Investigação em Ciências da Saúde: Enfermagem, Coimbra, Portugal.

**Keywords:** Self-Injurious Behavior, Mental Health Assistance, Adolescent, Psychiatric Nursing, Nursing Methodology Research, Health Human Resource Training

## Abstract

**Objective:**

to develop and validate educational material to strengthen adolescent health care on non-suicidal self-injury.

**Method:**

methodological research designed in three stages: (1) construction of the material based on a mixed study on needs related to the theme through social networks and an umbrella review on health care related to non-suicidal self-injury; (2) validation with 10 experts in mental health and/or self-inflicted violence selected through the Lattes Platform; (3) evaluation by the target public, with health professionals being invited, without restriction of training. Validation and evaluation data were collected by using a sociodemographic questionnaire and the Suitability Assessment of Materials for evaluation of health-related information for adults. We used descriptive statistics, content validity index, and Gwet’s AC1 test.

**Results:**

the material obtained good general acceptance and reliability in the validation by the experts (AC1= 0.633; p=0.0000) and in the evaluation by the target public (AC1=0.716; p=0.0000). All professionals pointed out the personal contribution and educational potential of the material.

**Conclusion:**

we highlight the construction of science-based educational material to strengthen the health care for adolescents with non-suicidal self-injury.

## Introduction

Non-suicidal self-injury (NSSI), popularly known as self-mutilation, is considered an intentional and self-directed behavior of aggression, without conscious intention of suicide and for reasons not accepted socially or culturally^1^ ^-^ ^4^. It is a multifactorial behavior that affects, in particular, adolescents aged 11 to 13 years and has important emotional, physical and social repercussions, in the short and long term^5^ ^-^ ^6^.

NSSI can have several functions or purposes and there is the possibility of coexistence of multiple functions^7^ ^-^ ^8^. Intrapersonal functions (associated with managing or altering an internal state) are more frequent than interpersonal functions (related to communicating problems or influencing the external environment)^8^. Such finding emphasizes the suffering related to the behavior and the importance of avoiding trivialization^7^.

Adolescents with NSSI report negative experiences such as misunderstanding, judgment and lack of empathy and credibility when seeking health care^9^ ^-^ ^10^. Health professionals also highlight the lack of formal training, governability and a feeling of unpreparedness to provide health care to adolescents with NSSI^11^. A Brazilian study conducted with health and education professionals observed the trivialization of NSSI, considered not relevant as a health issue^12^. Researchers also point out a restrictive care, without coordination in the follow-up, in addition to a superficial therapeutic approach and adherence problems^13^ ^-^ ^15^. Inadequate compulsory notification of cases of NSSI is also highlighted. The underreporting and incorrect completion of the intentionality of self-inflicted violence severely compromise the quality of the recording of this information^16^.

Studies point to the importance of investing in professional training and in the construction of guidelines that can guide the health care of adolescents with non-suicidal self-injury^11^ ^,^ ^17^. A Brazilian study with nursing undergraduates found the relation between reading educational materials and more positive attitudes about self-inflicted violence^18^. An Australian research highlighted education and training to improve professional knowledge and attitudes in Nursing, contributing to more positive results in the health care of adolescents who self-injure^19^.

In Brazil, the production of textual content on the behavior arises from 2018, most of which are educational booklets for the general public and addressing the behavior in the background. This study was based on the potential of the construction of textual material based on methodological research to support the education of health professionals for the enhancement of health care for adolescents with NSSI. Thus, the objective of this study was to develop and validate educational material to strengthen adolescent health care on non-suicidal self-injury.

## Method

**Figure 1:**
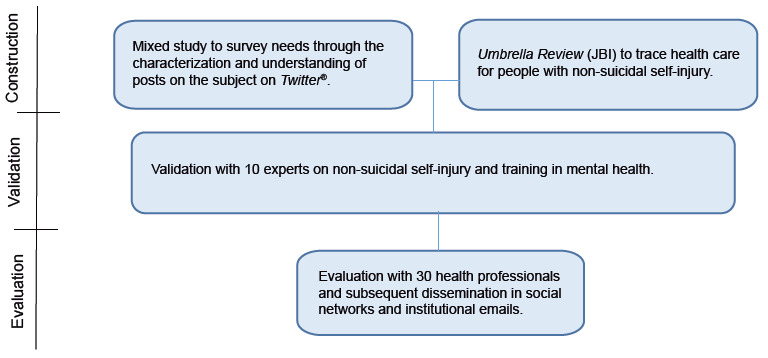
Design of methodological steps for development, validation and evaluation of the educational material. Ribeirão Preto, SP, Brazil, 2021

This is a methodological study for the construction of products with high methodological rigor, validated by experts and evaluated by the public for which it is intended^20^. This research meets the recommendations of the Consolidated criteria for Reporting Qualitative research (COREQ) and followed the steps described in Figure 1.

### Construction of educational material

The educational material was built based on two scientific studies, the first being mixed^21^ and focused on the survey of topics of interest and needs on the subject from posts on the social network *Twitter*. At first, we carried out a quantitative analysis of 6,302 public posts on NSSI, in Portuguese, from September 2016 to August 2017. Subsequently, we conducted thematic analysis^22^ of 663 posts with content encouraging the practice of NSSI. The results enabled us to characterize and trace subjects related to non-suicidal self-injury through a contemporary social discussion tool.

The second study consisted in an umbrella review (Joanna Briggs Institute)^23^ to trace best practices for prevention and professional health care for adolescents with NSSI. We conducted a qualitative analysis of a total of 73 reviews on NSSI published from 2011 to 2021. The results of the studies were disseminated in two scientific articles and guided, in conjunction with guidelines and legislation, the textual development of the content of the educational material. The graphic construction was supported by two Product Design scholars from a junior company of a public higher education institution, in addition to an illustrator.

### Validation and evaluation of educational material

The experts participating in the study were selected through the Lattes Platform, in April 2021, by means of two distinct searches with the terms “non-suicidal self-injury” and “training in mental health” and nationality filter “Brazilian.” The experts were selected according to the criteria of expertise^24^ and should meet at least one of the following criteria: (1) completed master’s or doctoral degree on the subject; (2) advisory of academic work on the area of interest; (3) experience in teaching in the area; (4) lectures given on the subject in some national or international scientific event. Experts who did not return to the evaluation within the stipulated period of 30 days were considered as having quit.

The target public (health professionals) was selected through the dissemination of the research in social networks and institutional e-mails. We invited higher education-level health professionals, without specification of training. Professionals who did not return to the evaluation within the stipulated period of 30 days were considered as having quit.

Data collection used a characterization questionnaire (age, gender, education, origin and area of experience: NSSI or training in mental health), in addition to an adapted version of the Suitability Assessment of Materials for evaluation of health-related information for adults (SAM)^25^.

SAM consists of 22 questions distributed in the following areas: (1) content, (2) literacy requirement, (3) illustrations, (4) layout and presentation, (5) stimulation/motivation for learning, and (6) cultural adequacy. In each item, the evaluation is performed by a three-point Likert scale (super adequate, adequate and inadequate), and the overall analysis or the isolated analysis of each item can be interpreted. The choice of the instrument is justified by its wide use in scientific studies, ease of understanding of the items and the time of application^25^.

Data collection was conducted in 2021 at two different times, with experts in July and August and health professionals in September. Data were collected through an explanatory message with a hyperlink to access Survey Monkey^®^, which provided the Informed Consent Form (ICF), the sociodemographic questionnaire, the educational material (for download), and the SAM instrument.

All data were organized and processed in Microsoft Excel 10 and subsequently processed and analyzed by STATA statistical software. Simple descriptive statistics was used for analysis of characterization data. For analysis of the educational material evaluation data, we decided to use the Content Validity Index (CVI) with acceptance level of 80%. The CVI measures the proportion of agreement on a certain aspect of the material under evaluation^26^.

We also used Gwet’s AC1 test, which measures the degree of agreement or the reliability of the agreement obtained between the evaluators. This test is robust, communicable, interpretable and not sensitive to marginal homogeneity, and it can be used with nominal and ordinal variables and with missing data^27^. To classify the reliability obtained, we adopted the following parameters: poor reliability (less than or equal to 0.20); fair (0.21 to 0.40); moderate (0.41 to 0.60); good (0.61 to 0.80) and very good (greater than 0.81)^27^.

The research was evaluated and approved by the Research Ethics Committee of the School of Nursing of Ribeirão Preto - University of São Paulo (CEP/EERP-USP) under opinion No. 3.627.052 and CAAE 16843419.9.0000.5393.

## Results

### Construction

The educational material “Non-suicidal self-injury: health care and mental health promotion” was composed of 58 pages, divided into pre-textual elements (cover, catalog sheet, epigraph, preface and summary), seven chapters that contained answers to frequent questions about the subject (definition, prevalence, risk groups), strengthening protective factors (emotional education, expansion of social education on NSSI and mental health, reduction of social vulnerabilities and strengthening of public policies), evaluation (reception, behavioral characteristics, risk and protection factors, motivations and functions, suicidal intentionality, support strategies and resources, internet and currentness) and professional follow-up (health care actions, compulsory and parental notification, socioemotional interventions, psychotherapy and pharmacology) for adolescents with NSSI, support network (family, school and internet), in addition to appendices (crisis management plan and framework for institutional plans for prevention of NSSI).

The educational material was originally distributed free of charge in digital version through the Editorial Support Center (CAEd/EERP-USP) and the InspirAção website (www.inspiracao-leps.com.br). Learn more about the material: http://www.eerp.usp.br/caed/ebook/6/

### Validation by experts

The educational material validation stage had participation of ten experts, most of them female (70%), with a mean age of 51.5 years (SD=15.48, 29-68 years) residing in the Southeast region (70%) in the states of Rio de Janeiro (30%), São Paulo (20%) and Minas Gerais (20%). Regarding academic training, there were four psychologists, three nurses, two physicians, and one social worker. The majority had a complete doctorate (90%), with experience in the subject of non-suicidal self-injury (90%) or training in mental health (90%).

Regarding the acceptance and agreement of the items of the educational material, all items reached the minimum approval criterion (CVI≥80%) (calculated from the sums of adequate and regular responses). Most items related to purpose, content, context, reading level, illustrations, motivation and self-efficacy were evaluated at 100% adequacy (CVI=1.0) (Table 1).

**Table 1:** Acceptance and agreement with the items of the educational material in the stage of validation by experts (n=10). Ribeirão Preto, SP, Brazil, 2021

Item	Agreement		CVI^†^
	Yes n(%)	No n(%)	
Evidence of purpose	100	0	1.0
Content deals with behaviors	100	0	1.0
Purpose-Focused Content	100	0	1.0
Content highlights key points	100	0	1.0
Reading level requirement	80*	0	1.0
Writing in the active voice	100	0	1.0
Common vocabularies in the text	80	20	0.8
Context before information	90	10	0.9
Facilitation of learning by topics	100	0	1.0
Purpose of the illustration is clear	100	0	1.0
Types of the illustrations	100	0	1.0
Illustrations are relevant	100	00	1.0
Lists and tables have explanations	90	10	0.9
Captions in the illustrations	80*	0	1.0
Layout characteristic	100	0	1.0
Font size and type	100	0	1.0
Use of subtitles	100	0	1.0
Specific instructions with examples	90	10	0.9
Motivation and self-efficacy	100	0	1.0
Uses interaction	100	0	1.0
Consistent with logic, language and experience	90	10	0.9
Cultural image and examples	100	0	1.0

*Item with blank answers or non-applicable (N/A); ^†^Content Validity Index

Regarding the reliability of agreement, the educational material showed good reliability in the overall evaluation. The items on content and motivation showed very good reliability. The illustrations, layout and culture showed good reliability. And the item language presented a moderate reliability (Table 2).

**Table 2:** Reliability of the agreement of the educational material validation by experts (n=10). Ribeirão Preto, SP, Brazil, 2021

	AC1*	SD^†^	95%CI^‡^	P-value
General	0.633	0.082	(0.448,0.819)	0.0000
Content	1.000	0.000	(1,1)	0.0000
Language	0.587	0.153	(0.242,0.933)	0.0039
Illustrations	0.778	0.094	(0.566,0.99)	0.0000
*Layout*	0.672	0.205	(0.207,1)	0.0097
Motivation	0.819	0.124	(0.538,1)	0.0001
Culture	0.615	0.265	(0.016,1)	0.0452

*Statistical value; ^†^Standard Deviation; ^‡^Confidence Interval

The main suggestions were related to three general themes: (1) clarity of vocabulary (use of vocabulary and clarification of less common terms), (2) NSSI and health care (operational definition of NSSI, notification and mention of minority groups), and (3) editing (grammar revision and examples). Even reaching the minimum expected acceptance (CVI=0.8), changes were made in the clarification of less usual terms and implications of the association of the Child Protective Services for better understanding.

Regarding the educational material, the experts highlighted the importance of the content for the health care of adolescents with NSSI: *The content highlights the important points for NSSI (in relation to the adolescent and family)* (A2). In addition to important information for the support network (family, school): *The content is very useful for people who want to have information about NSSI and what procedures are necessary* (A6).

### Evaluation by the target public

At this stage, 75 health professionals agreed to participate in the research to evaluate the educational material. However, 30 health professionals (40%) completed the evaluation of the educational material. Most professionals were female (90%), white (80%), with a mean age of 36.9 years (SD=13.1, 23-68 years) residing in the Southeast region (96.7%) in the states of São Paulo (80%), Minas Gerais (16.7%) and Rio de Janeiro (3.3%). Ribeirão Preto (SP) was the city with the most participants (46.7%), followed by Divinópolis (MG) (10.0%) and São Paulo (SP) (6.7%).

Most professionals had a degree in Nursing (60.0%) and a master’s degree (43.3%). Most reported lacking previous training on non-suicidal self-injury (73.3%), but having had professional experience related to the subject (63.3%).

All items of the educational material reached the minimum approval criteria (CVI≥80%) in the evaluation by health professionals. All items reached validity of acceptance of health professionals above 90% (Table 3).

**Table 3:** Acceptance and agreement with the items in the stage of evaluation of the material by the target public (n=30). Ribeirão Preto, SP, Brazil, 2021

Item	Agreement		CVI^†^
	Yes n(%)	No n(%)	
Evidence of purpose	96.7	3.3	0.97
Content deals with behaviors	93.3	6.7	0.93
Content focused on the purpose	96.7	3.3	0.97
Content highlights key points	96.7	3.3	0.97
Reading level requirement	90.0*	3.3	0.96
Writing in the active voice	100	0	1.0
Common vocabularies in the text	96.7	3.3	0.97
Context before information	100	0	1.0
Facilitation of learning by topics	100	0	1.0
Purpose of the illustration is clear	96.7	3.3	0.97
Types of the illustrations	100	0	1.0
Illustrations are relevant	96.7*	0	1.0
Lists and tables have explanations	100	0	1.0
Captions in the illustrations	90*	10	1.0
Layout characteristic	100	0	1.0
Font size and type	100	0	1.0
Use of subtitles	100	0	1.0
Specific instructions with examples	100	100	1.0
Uses interaction	90*	6.7	0.93
Motivation and self-efficacy	96.7	3.3	0.93
Consistent with logic, language and experience	100	0	1.0
Cultural image and examples	100	0	1.0

^*^Item with blank answers or non-applicable (N/A); ^†^Content Validity Index

Regarding the reliability of agreement, the educational material showed good reliability in the overall evaluation. The items on language, illustrations and motivation showed good reliability. And the items on content, layout and culture showed very good reliability (Table 4).

**Table 4:** Reliability of the agreement of the evaluation by the target public (n=30) of the educational material. Ribeirão Preto, SP, Brazil, 2021

	AC1*	SD ^†^	95%CI ^‡^	P-value
General	0.716	0.053	(0.609,0.824)	0.0000
Content	0.881	0.054	(0.771,0.991)	0.0000
Language	0.754	0.068	(0.615,0.892)	0.0000
Illustrations	0.769	0.063	(0.641,0.898)	0.0000
Layout	0.879	0.063	(0.751,1)	0.0000
Motivation	0.705	0.086	(0.529,0.881)	0.0000
Culture	0.891	0.079	(0.729,1)	0.0000

*Statistical value; ^†^Standard Deviation; ^‡^Confidence Interval

Among the suggestions, the professionals indicated characteristics of the layout, such as the use of more images and infographics, the reading level requirement, and the spelling and grammar revision. The suggestions related to the topics were accepted; however, major changes in the diagramming were not made because they reached the minimum acceptance criteria in the evaluation. The health professionals highlighted the design and *layout* of the material in facilitating the process of reading and understanding information, such as: *the formatting and diagramming of the text make it easy and pleasant, the illustrations are very beautiful and contribute not only to embellish the material, but also to emphasize the emotional aspects of the content discussed in the text* (P7) *and congratulations on the graphic part of the text. Attractive and motivating* (P18).

The reading level requirement was well evaluated and the suggestions highlighted the complexity: *the text requires a good level of literacy, but the authors properly used the common words to elucidate complex issues* (P18), but emphasizing the adequacy for the target public: *meets the cultural profile of health professionals in Brazil* (P18)*.* The health professionals highlighted the content, organization and insertion of examples, tips and suggestions of other supplementary materials: *The use of topics facilitates the reading, creating dynamism in what is presented* (P13) *and the booklet addresses concepts that promote the understanding of the theme in an organized manner, I point out as positive the examples of management and interaction* (P23).

All participants (100%) affirmed about the personal contribution of the educational material with new knowledge on the theme and in the reception of adolescents with NSSI. All participants (100%) affirmed that the educational material has the potential to contribute to the education of health professionals for the reception of adolescents with NSSI.

## Discussion

The educational material characterizes the phenomenon of NSSI and addresses useful knowledge for health care through interprofessional and intersectoral follow-up. The experts and professionals made a positive evaluation of the material and reliability was observed in the agreement of the participants. All professionals highlighted that reading provided a personal contribution and considered that the material can contribute to professional education for the reception and health care of adolescents with NSSI.

Quality health care for people with NSSI requires understanding their needs, individual preferences, risk and protection factors to which they are exposed, as well as the context and culture in which the individual is situated. Adolescents should be encouraged to respect their own limits, communicate how they want to be helped, recognize symptoms early and learn about ways to seek help and actively participate in their own health care. In nursing care, it may also be important to address the assessment and attention to physical needs, motivation for the adoption of healthy behaviors, knowledge of and access to rights guaranteed by law, tracing, notification and support for situations involving risk of violence, among others. There is a wide variety of possible actions depending on each situation. Thus, it is important to establish customized and achievable goals that are updated periodically. All these elements were addressed in the material constructed and the entire production of the material was based on national^28^ and international^29^ recommendations on the subject and was guided by assumptions related to the defense of health promotion, community-based, with social participation and founded on human rights.

In health care actions related to NSSI, it is essential to invest in the strengthening of protective factors, such as self-knowledge and assertive expression of needs, resilience, emotional regulation, self-esteem and self-efficacy, hope, healthy lifestyle, coping strategies, problem-solving skills, satisfactory interpersonal relationships, and support network^13^ ^-^ ^14^ ^,^ ^28^ ^-^ ^29^.

It is noted that the material promotes expanded understanding of NSSI as a multifactorial phenomenon, which involves not only individual and relational factors, but also several social health determinants. Thus, we highlight the need to guarantee human rights and mental health^29^ and to fight for decent living conditions and quality of life. Such aspects are particularly important in the current Brazilian conjuncture, with increased poverty, food insecurity^30^, school dropout rate^31^, easier access to lethal methods^32^ ^-^ ^33^, social and environmental injustice^34^ and disruption of social, health care and, especially, mental health programs^35^.

Despite the approval of a national policy geared toward the prevention of NSSI represents an advance^36^ ^-^ ^38^, for it to translate into favorable results, it is still necessary to consolidate public policies that subsidize conditions for a dignified life and access to quality mental health care. Some ways to achieve these results are the commitment to human rights, social participation, expansion of access to mental health care services^39^, investment in science, and governmental commitment.

Another issue to be discussed is health training for NSSI-related prevention and care. In this study, most professionals highlighted the lack of formal training for provision of health care to adolescents with NSSI. However, the participants stated that they had professional experience in the subject. Thus, it is necessary to expand and enhance the professional training and qualification for the prevention of NSSI. It is important to invest in the construction of new teaching-learning strategies, but also in the inclusion of this content in the curricula of health care-related programs.

In the process of constructing the material, the predominant use of international scientific literature is highlighted. There were gaps in the contextualization of NSSI in socially vulnerable groups (indigenous people, black people, LGBTQIA+, quilombolas, among others). These aspects evince the need to invest in diversified scientific research on the phenomenon in different contexts and social groups in Brazil.

The literature contains few indications of recommendations of NSSI-related health care that are associated with the virtual environment. It is necessary to conduct research on best practices and guidelines for health care actions that consider self-inflicted violence and the safe use of screens^40^ ^-^ ^41^.

The educational material was disseminated via institutional e-mail to Universities, State Health Secretariats, governmental and non-governmental agencies. The content was also disseminated on social networks of the research group (@inspiracaoleps).

The study presents limitations related to the restriction of the validation process in the Southeast region. However, this study presents the first Brazilian educational material produced through methodological research with a focus on training for the prevention of NSSI. It is expected that the material will contribute to enhance interprofessional health care and raise new discussions, expanding perspectives and possibilities of health care for adolescents with NSSI.

## Conclusion

Methodological research enabled us to construct educational material to promote the enhancement of health care on NSSI. The results of the validation and evaluation (by experts and target public, respectively) highlight the adequacy of the content for the reality of Brazilian health professionals and with the possibility of addressing gaps traced in the academic training in health care about NSSI. The educational material, validated by experts and the target public, has the potential to contribute to professional training actions for the enhancement of health care for adolescents with NSSI.
